# Supplementary material for: The interactome of intact mitochondria by cross-linking mass spectrometry provides evidence for coexisting respiratory supercomplexes
Source: Mol Cell Proteomics. 2017 Dec 8;17(2):216–32. doi: 10.1074/mcp.RA117.000470 (PMC5795388; doi:10.1074/mcp.RA117.000470)
Supplement: Supplemental Data [file supp_RA117.000470_133922_0_supp_23978_fzffwf.zip › spectra_annotation/mito_DR_spectra_annotation/72-1-6-1-1-1.pdf]

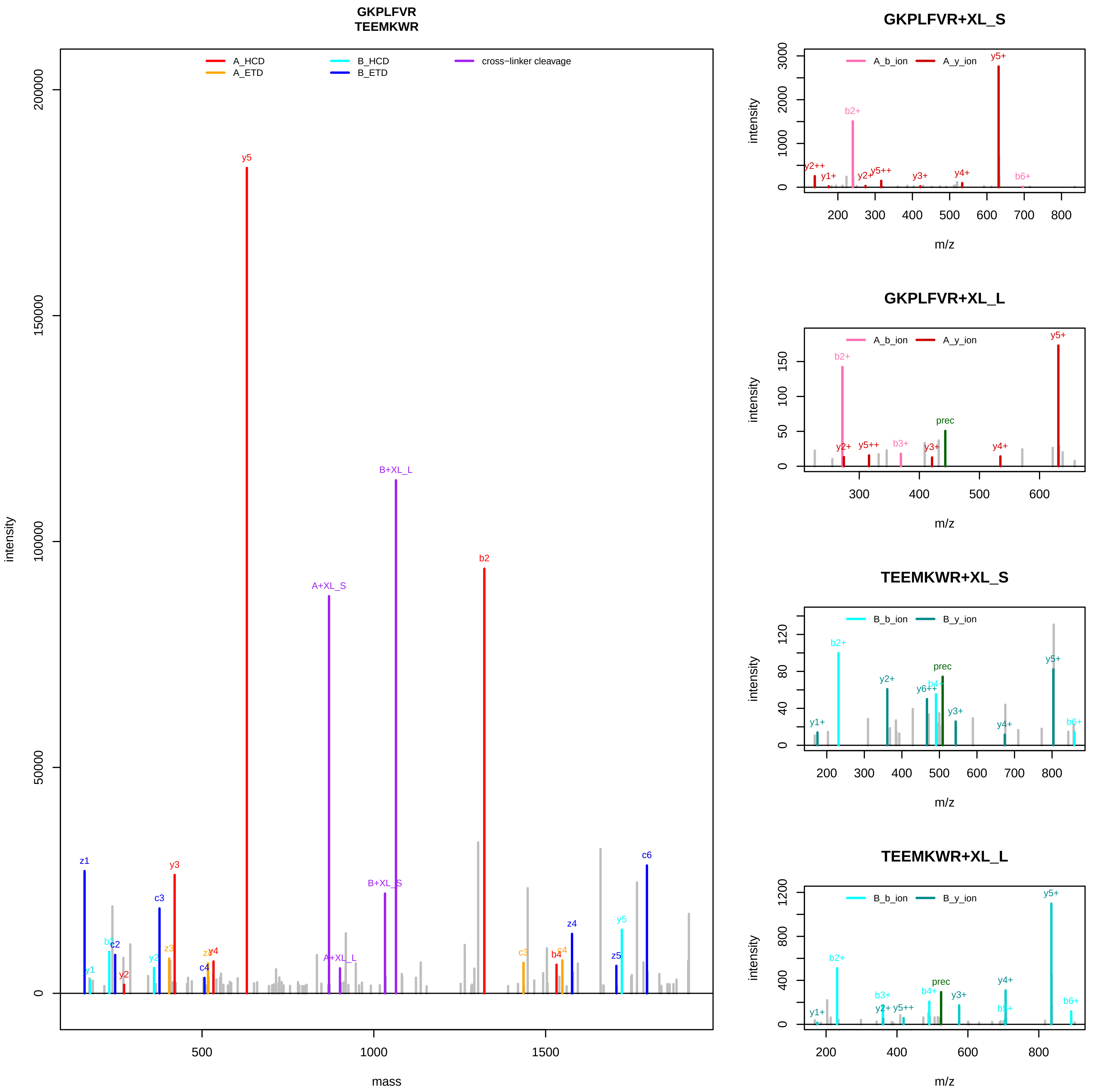

GKPLFVR
TEEMKWR
A_HCD
A_ETD
B_HCD
B_ETD
cross−linker cleavage
y5
B+XL_L
A+XL_S
b2
B+XL_S
A+XL_L
z1
c3
y3
c6
z4
y5
z5
c4
z3
y4
c3
b4
c4
intensity
mass
GKPLFVR+XL_S
A_b_ion
A_y_ion
y5+
b2+
y2++
y1+
y2+
y5++
y3+
y4+
b6+
m/z
GKPLFVR+XL_L
prec
b3+
TEEMKWR+XL_S
B_b_ion
B_y_ion
y6++
b4+
TEEMKWR+XL_L
